# Supplementary material for: Hepatotoxicity in Carp (Carassius auratus) Exposed to Perfluorooctane Sulfonate (PFOS): Integrative Histopathology and Transcriptomics Analysis
Source: Animals (Basel). 2025 Feb 19;15(4):610. doi: 10.3390/ani15040610 (PMC11851982; doi:10.3390/ani15040610)
Supplement: Supplementary file 1 [file animals-15-00610-s001.zip › animals-3389860-supplementary.pdf]

# Supporting information

# Hepatotoxicity in carp (*Carassius auratus*) exposed to perfluorooctane sulfonate (PFOS): Integrative histopathology and transcriptomics analysis

Lin Tang[1,†], Guijie Hao[2,†], Dongren Zhou[2], Yunpeng Fan[2], Zihao Wei[1], Dongsheng Li[1], Yafang Shen[2], Haoyu Fang[1], Feng Lin[2], Meirong Zhao[1], Haiqi Zhang[2*]

[1] Key Laboratory of Microbial Technology for Industrial Pollution Control of Zhejiang Province, College of Environment, Zhejiang University of Technology, Hangzhou, 310014 Zhejiang, China

[2] Key Laboratory of Freshwater Fisheries Healthy Aquaculture, Ministry of Agriculture and Rural Affairs, Key Laboratory of Fish Health and Nutrition of Zhejiang Province, Key Laboratory of Fishery Environment and Aquatic Product Quality and Safety of Huzhou City, Zhejiang Institute of Freshwater Fisheries, Huzhou, 313001 Zhejiang, China

[†]These authors contributed equally to this work.

***Corresponding author:**

Haiqi Zhang (E-mail: zmk407@126.com, Tel: (+86)13819493421)

# Contents

**Table S1.** RIN values for each individual sample.

**Table S2.** Primers for qRT-PCR.

**Table S3.** $LC_{50}$ values for Carps exposed to PFOS for 96 h.

**Figure S1.** The fitting curve of 96 h $LC_{50}$. Crucian carp were treated with 0, 10, 15, 20, 25, 30, and 35 mg/L of PFOS at a density of 15 fish per tank for 96 h. Data were collected from three independent assays.

**Supplemental Materials**

**Table S1.** RIN values for each individual sample.

| sample name | Concentration (ng/μL) | volumetric (μL) | overall amount (ug) | RIN |
|---|---|---|---|---|
| C1 | 1112.4 | 40 | 46.50 | 6.4 |
| C2 | 1454.5 | 40 | 58.18 | 6.3 |
| C3 | 1360.9 | 40 | 54.44 | 6.0 |
| L1 | 1553.6 | 40 | 62.12 | 5.8 |
| L2 | 1231.4 | 40 | 49.26 | 5.8 |
| L3 | 1215.3 | 40 | 48.61 | 5.9 |
| M1 | 1674.7 | 40 | 66.99 | 5.4 |
| M2 | 1194.5 | 40 | 47.78 | 6.8 |
| M3 | 1203.2 | 40 | 48.13 | 6.7 |
| H1 | 1731.3 | 40 | 69.25 | 5.4 |
| H2 | 1365.9 | 40 | 54.64 | 5.5 |
| H3 | 1187.4 | 40 | 47.50 | 5.6 |

**Table S2.** Primers for qRT-PCR.

| NCBI numbers | gene name | Primer | Sequence (5′-3') |
|---|---|---|---|
| *113039597* | *ODF3L2-X3* | F | GCTATCGGCTTTGTAGGCCA |
| | | R | GGGTGGAGTAGATTGCTGCT |
| *113042045* | *COX6B1* | F | ATCGCTGCCAAAAAGCACTG |
| | | R | GGGGTGAGACGATACCACAG |
| *113044583* | *CPO* | F | ATGTGAAGAAGCGTACCGGG |
| | | R | CCAGGTGGTTTTTGTTAGACAGT |
| *113049645* | *LIMP II* | F | GGAGAACACCTCCGTTCCTG |
| | | R | ACTGGAGAAGCTCCCGGTAT |
| *113050556* | *ANGPTL3* | F | CAATCCGAGGGGTCACACAA |
| | | R | CCGCCATGGGTCTTTACCAT |
| *113056608* | *TMEM254* | F | GTGTCCCAGAGCACACTGAC |
| | | R | CTGTAACCCGTCTCTGAGCA |
| *113062105* | *NKATP1BP1* | F | AGTGTCCATAGCCGCATGAT |
| | | R | CCGGCTCTTTGTCAACATCG |
| *113067742* | *CYP2K1* | F | GTTGTAGTAAGGGCTATTCTTGTG |
| | | R | GTGCATGCTCCGTGAAAGAG |
| / | *Pir* | F | TCACCGTGGATTTGAGACGG |
| | | R | ATGAATGCGTCCATCCGACA |
| *113045050* | *BASP1* | F | GCAGACGCCTGAGAAAGGA |
| | | R | AGGTGTCATTGGTTGTCTCGG |
| *113110911* | *GPX1* | F | CTGAAGTACGTCCGTCCTGG |
| | | R | GGATCCCCCATCAAGGACAC |
| / | *β-actin* | F | CGAGAAGATGACCCAGATCA |
| | | R | GATCTTCATGAGGTAGTCAG |

**Table S3.** $LC_{50}$ values for Carps exposed to PFOS for 96 h.

| Time / h | regression equation | $R^2$ | $LC_{50}$（mg/L） | 95% CI (mg /L) |
|---|---|---|---|---|
| 24 | y=0.099x-4.103 | 0.848 | 41.409 | 38.50 - 45.13 |
| 36 | y=0.116x-3.381 | 0.987 | 29.263 | 27.34 - 32.01 |
| 72 | y=0.136x-3.378 | 0.925 | 24.917 | 21.12 - 28.26 |
| 96 | y=0.164x-3.801 | 0.765 | 23.166 | 18.60 - 26.18 |

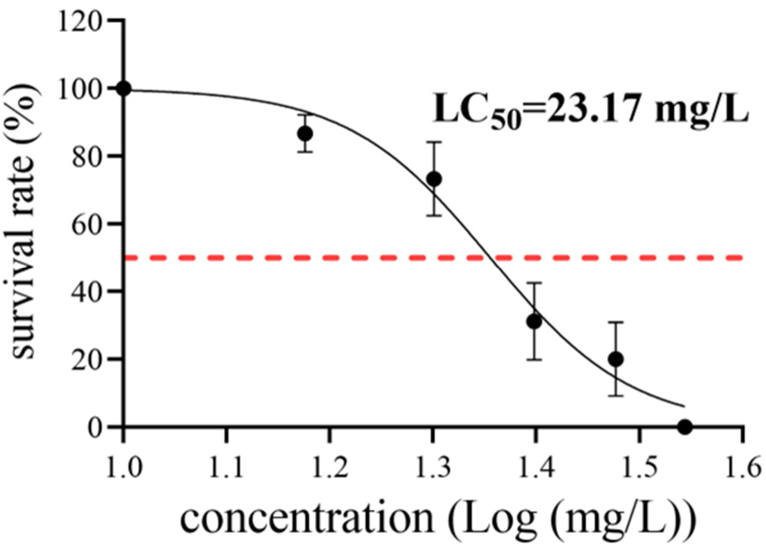


**Figure S1.** The fitting curve of 96 h $LC_{50}$. Crucian carp were treated with 0, 10, 15, 20, 25, 30, and 35 mg/L of PFOS at a density of 15 fish per tank for 96 h. Data were collected from three independent assays.
